# Reliability, stability during long-term storage, and intra-individual variation of circulating levels of osteopontin, osteoprotegerin, vascular endothelial growth factor-A, and interleukin-17A

**DOI:** 10.1186/s40001-024-01722-w

**Published:** 2024-02-17

**Authors:** Tomoki Nakamizo, John Cologne, Takeshi Kishi, Tetsuya Takahashi, Mayumi Inoue, Hiroyuki Ryukaku, Tomonori Hayashi, Yoichiro Kusunoki, Saeko Fujiwara, Waka Ohishi

**Affiliations:** 1 Department of Clinical Studies, RERF, Nagasaki, Japan; 2 Department of Statistics, RERF, Nagasaki, Japan; 3 Division of Clinical Laboratories, RERF, Hiroshima, Japan; 4 Faculty of Rehabilitation, Hiroshima International University, Hiroshima, Japan; 5 Department of Molecular Biosciences, RERF, Hiroshima, Japan; 6 Faculty of Pharmacy, Yasuda Women’s University, Hiroshima, Japan; 7 Department of Clinical Studies, RERF, Hiroshima, Japan

**Keywords:** Molecular epidemiology, Biomarkers, Cytokines, Biological variation, Individual, Reproducibility of results, Measurement accuracy

## Abstract

**Background:**

Studies in many populations have reported associations between circulating cytokine levels and various physiological or pathological conditions. However, the reliability of cytokine measurements in population studies, which measure cytokines in multiple assays over a prolonged period, has not been adequately examined; nor has stability during sample storage or intra-individual variation been assessed.

**Methods:**

We assessed (1) analytical reliability in short- and long-term repeated measurements; (2) stability and analytical reliability during long-term sample storage, and (3) variability within individuals over seasons, of four cytokines—osteopontin (OPN), osteoprotegerin (OPG), vascular endothelial growth factor-A (VEGF-A), and interleukin-17A (IL-17A). Measurements in plasma or serum samples were made with commercial kits according to standard procedures. Estimation was performed by fitting a random or mixed effects linear model on the log scale.

**Results:**

In repeated assays over a short period, OPN, OPG, and VEGF-A had acceptable reliability, with intra- and inter-assay coefficients of variation (CV) less than 0.11. Reliability of IL-17A was poor, with inter- and intra-assay CV 0.85 and 0.43, respectively. During long-term storage, OPG significantly decayed (− 33% per year; 95% confidence interval [− 54, − 3.7]), but not OPN or VEGF-A (− 0.3% or − 6.3% per year, respectively). Intra- and inter-assay CV over a long period were comparable to that in a short period except for a slight increase in inter-assay CV of VEGF-A. Within-individual variation was small for OPN and VEGF-A, with intra-class correlations (ICC) 0.68 and 0.83, respectively, but large for OPG (ICC 0.11).

**Conclusions:**

We conclude that OPN and VEGF-A can be reliably measured in a large population, that IL-17A is suitable only for small experiments, and that OPG should be assessed with caution due to degradation during storage and intra-individual variation. The overall results of our study illustrate the need for validation under relevant conditions when measuring circulating cytokines in population studies.

**Supplementary Information:**

The online version contains supplementary material available at 10.1186/s40001-024-01722-w.

## Background

Cytokines, including osteoprotegerin (OPG), osteopontin (OPN), vascular endothelial growth factor-A (VEGF-A), and interleukin-17A (IL-17A), are implicated in the pathogenesis of disorders such as cancer and cardiovascular diseases [1–9]. Many studies reported associations of circulating cytokine levels with various diseases [3, 6, 10–13], suggesting pathogenetic and diagnostic roles. However, there was considerable heterogeneity among studies, with some disputing the associations [3, 6, 11, 13]. For example, serum OPG level was or was not associated with cardiovascular event risk [13] and was associated with either better or worse prognosis with breast cancer [3].

Such association or lack thereof should be interpreted in light of measurement reliability (intra- and inter-assay variability) and intra-individual variation, because large variations can mask differences between healthy and ill individuals [14]. An indicator of assay reliability, usually the coefficient of variation (CV), is typically provided by manufacturers, but the CV is often obtained from ideal small-scale experiments that differ from the real world of population studies, which measure many samples in multiple assays over an extended period. Because multiplicity could introduce additional variability, it is recommended that investigators of biomarker studies validate reliability under conditions that are as close as possible to those of their actual study [14]. Many studies, however, have not addressed potential differences in the assay environment, merely reporting the manufacturer-provided CV as given and leaving it at that.

Furthermore, specimens in population studies are often stored for a long time before measurement, raising concerns about degradation during sample storage. However, little is known about this potential source of non-pathological variation in the inferred circulating levels of cytokines. As far as we are aware, few studies have investigated long-term stability or intra-individual variation of VEGF-A [15–18], IL-17A [15, 16], or OPG [19], and none have done so with OPN.

In this study, we aimed to evaluate reliability of measurement, stability during long-term storage, and variation within individuals of levels, associated with plasma OPN and serum OPG, IL-17A, and VEGF-A. This study is a part of research on atherosclerosis conducted in 2010–2014 as part of the Adult Health Study (AHS) of atomic bomb survivors at the Radiation Effects Research Foundation (RERF) in Hiroshima and Nagasaki, Japan (RERF Research Protocol RP2-11). The cytokines assessed were selected according to our hypothesis on the pathogenesis of atherosclerosis.

## Methods

### Measurement

Plasma OPN was measured with an enzyme-linked immunosorbent assay (ELISA) using Quantikine Human Osteopontin™ (R&D Systems, MN, USA) after 25-fold dilution according to the standard procedure implemented through an automated apparatus, EP-one (Sanko Junyaku, Tokyo, Japan). Serum IL-17A and VEGF-A were measured simultaneously with an immunofluorescence multiplex assay using Bio-Plex Pro Human Cytokine Standard 27-Plex, GroupI™ (Bio-Rad Laboratories, CA, USA) after fourfold dilution according to the standard procedure. Fluorescence was measured and analyzed on a Luminex platform (LUMINEX CORPORATION, TX, USA) to calculate concentration. Serum OPG was measured with an immunofluorescence multiplex assay using a Milliplex MAP Kit HumanBonePanel1A™ (Millipore Corporation, MA, USA) after 2- or 4-fold dilution according to the standard procedure with fluorescence analyzed on the Luminex platform. All assays were conducted in batches on 96-well plates with some wells allocated to standards for calibration, negative controls, and positive controls of known concentrations. Typical measurable ranges on the natural log scale were 2.1–6.2 for OPN (ng/mL), − 0.21–8.1 for VEGF-A (pg/mL), − 0.073–9.0 for IL-17A (pg/mL), and 2.4–11 for OPG (pg/mL).

### Assay of reliability over a brief period

To assess variation within and between assays over a brief period, we assayed a single sample on five to six quasi-consecutive days, with four or more measurements made in each assay. In the assays, we utilized anonymized residual samples from health exam participants and employee health checkups. The use of residual samples and publication of results based on them was approved by the institutional review board of RERF.

### OPN

A sample was created by pooling residual plasmas from health exam participants and employee checkups; plasmas were prepared by centrifuging peripheral blood in EDTA tubes immediately after drawing. The pooled sample was divided into five subsamples and stored at − 80 °C until time of measurement. The samples were measured on five quasi-consecutive days starting on the fifth day after preparation. On each day, a subsample was thawed at room temperature for 30 min, divided into four or five aliquots (depending on the sample volume), and measured in a batch.

### VEGF-A and IL-17A

Residual serum from a health exam participant, prepared by centrifuging peripheral blood in a serum separator tube 20 min after drawing, was divided into six subsamples and stored at − 80 °C until measured; measurements were made on six quasi-consecutive days, starting three months after preparation. On each day, a subsample was thawed at 4 °C for 15 h, divided into 15 aliquots, and measured in a batch. All experiments were conducted using the same kit, reagent lot, and platform.

### OPG

Residual serum from a health exam participant, prepared by centrifuging peripheral blood in a serum separator tube 20 min after drawing, was divided into six subsamples and stored at − 80 °C until measured; measurements were made on six quasi-consecutive days starting three months after preparation. On each day, a subsample was thawed at 4 °C for 2 h, divided into eight or nine aliquots, and measured in a batch. All experiments were conducted using the same kit, reagent lot, and platform.

### Assay for stability during long-term storage

To assess the variation within and between assays over a long period of storage, we measured aliquots from a single pooled sample stored for up to 44 months. This duration can accommodate additional potential sources of variation, including ambient temperature, multiple assay reagents, and potential drift in assay procedure.

Residual plasma or serum samples from health examinations were stored at − 80 °C. Two weeks later, the samples were thawed and pooled into a single plasma or serum sample. Each pooled sample was then divided into subsamples and stored again at − 80 °C. The stored subsamples were serially removed from storage and measured on 8 or 9 occasions over up to 44 months. For each measurement, a subsample was thawed, divided into five aliquots, and measured in a batch. During the 44-month experimental period, the assay environment for OPG, VEGF-A, and IL-17A was slightly altered because of manufacturer updates to the platform or assay kits. The platform software for OPG, VEGF-A, and IL-17A was updated from DNASISPlex™ to Luminex xPONENT™ at the time of the fourth assay (18th or 19th month), and the kit for OPG was updated from Luminex xMAP™ polystyrene beads to Luminex xMAP™ magnetic beads at the time of the sixth assay (33rd month). Because the software does not affect the assay reaction, we did not anticipate any effects of the software update on the measurement. Indeed, we confirmed comparability between software versions with VEGF-A (Additional file 1: Figure S1). However, we could not perform experiments to confirm inter-software comparability for IL-17A and OPG because of personnel and time constraints. Comparison between kits, on the other hand, suggested a difference in values of OPG (Additional file 1: Figure S2), so we adjusted for kit difference in the analysis. The assay environment for OPN remained the same during the entire period.

### Assay for intra-individual variation

To assess intra-individual variation, we measured the cytokine levels of healthy volunteers in four seasons over a year (July, October, January, and April). Plasma or serum samples were collected from 38 healthy volunteers aged 25 to 64 years (24 men and 14 women) with written consent. All samples were stored at − 80 °C and measured after about 60 months from the initial sampling in two batches consisting of the first and last two seasons, respectively. All assays were conducted using the same software, kits, and lots.

### Statistical analysis

All values were transformed by taking natural logarithms to achieve approximate normality.

We assessed variability within and between assays by calculating coefficients of variation (CV) defined as the standard deviation divided by the mean on the original scale. We estimated intra- and inter-assay CV by fitting a linear random effects model treating assay as a random effect. CV was calculated as $\sqrt{{\text{exp}}\left({\widehat{\sigma }}^{2}\right)-1}$, where ${\widehat{\sigma }}^{2}$ is the restricted maximum likelihood estimate of the variance component on the log scale; the same formula was applied to the Wald-type 95% confidence bounds on the variance component to obtain confidence bounds on the CV. Reliability was judged according to conventional criteria: good (< 0.15), fair (0.15–0.20), or poor (> 0.20) [14].

We evaluated stability during storage with intra- and inter-assay CV over the long term by fitting a linear mixed effects model treating storage time as a fixed effect and assay as a random effect. Decay with storage was expressed as percent change per year on the original scale calculated as 100*exp(*β*), where *β* stands for the coefficient of storage time in years from the model fit on the log scale. We also included experimental environment (combination of kit and software) as a fixed effect for OPG, VEGF-A, and IL-17A.

We evaluated intra-individual variation by calculating the intra-class correlation (ICC) with the fit of a linear random effects model treating individual donors as a random effect. The intra-individual variation was judged according to the conventional criteria [20]: excellent (> 0.75), good (0.60–0.75), fair (0.40–0.59), or poor (< 0.40).

## Results

### Measurement reliability over a short period

**Fig. 1:**
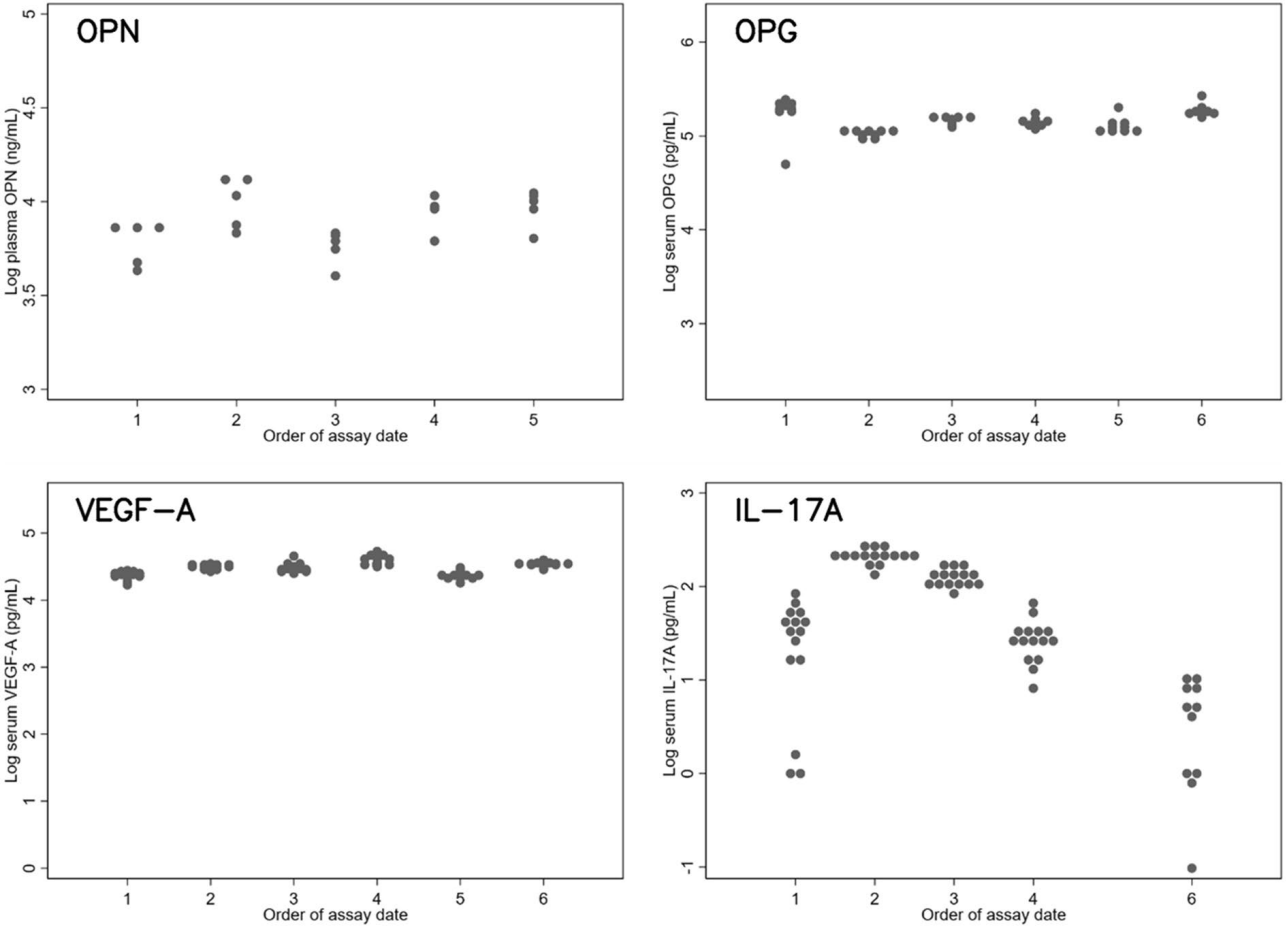
Distributions of cytokine values (log-transformed) in repeated assays over a brief period. OPG: A value below the limit of detection in the first assay was excluded. IL-17A: values below the limit of detection (all measurements in the fifth assay and four of the 15 measurements in the sixth assay) were excluded. The y-axes, except for IL-17A, are scaled to the ranges from the healthy volunteer measurements

Intra- and inter-assay CVs were good for OPN, OPG, and VEGF-A (Table 1). With IL-17A, all 15 measurements in the fifth assay and four of the 15 measurements in the sixth assay fell at or below the limit of detection. Even after those possible outliers were excluded, both inter- and intra-assay CVs, especially the former, were still large. These findings are illustrated in the distribution plots (Fig. 1).

**Table 1:** Measurement reliability over a short period

	Intra-assay CV [95% CI]	Inter-assay CV [95% CI]
OPN	0.11 [0.079, 0.15]	0.099 [0.042, 0.24]
OPG^*^	0.056 [0.046, 0.069]	0.10 [0.053, 0.19]
VEGF-A	0.055 [0.047, 0.064]	0.088 [0.047, 0.17]
IL-17A^*^	0.43 [0.36, 0.52]	0.85 [0.37, 2.91]

*Excluding the measurements below the limit of detection as in Fig. 1

### Stability and measurement reliability during long-term storage

**Fig. 2:**
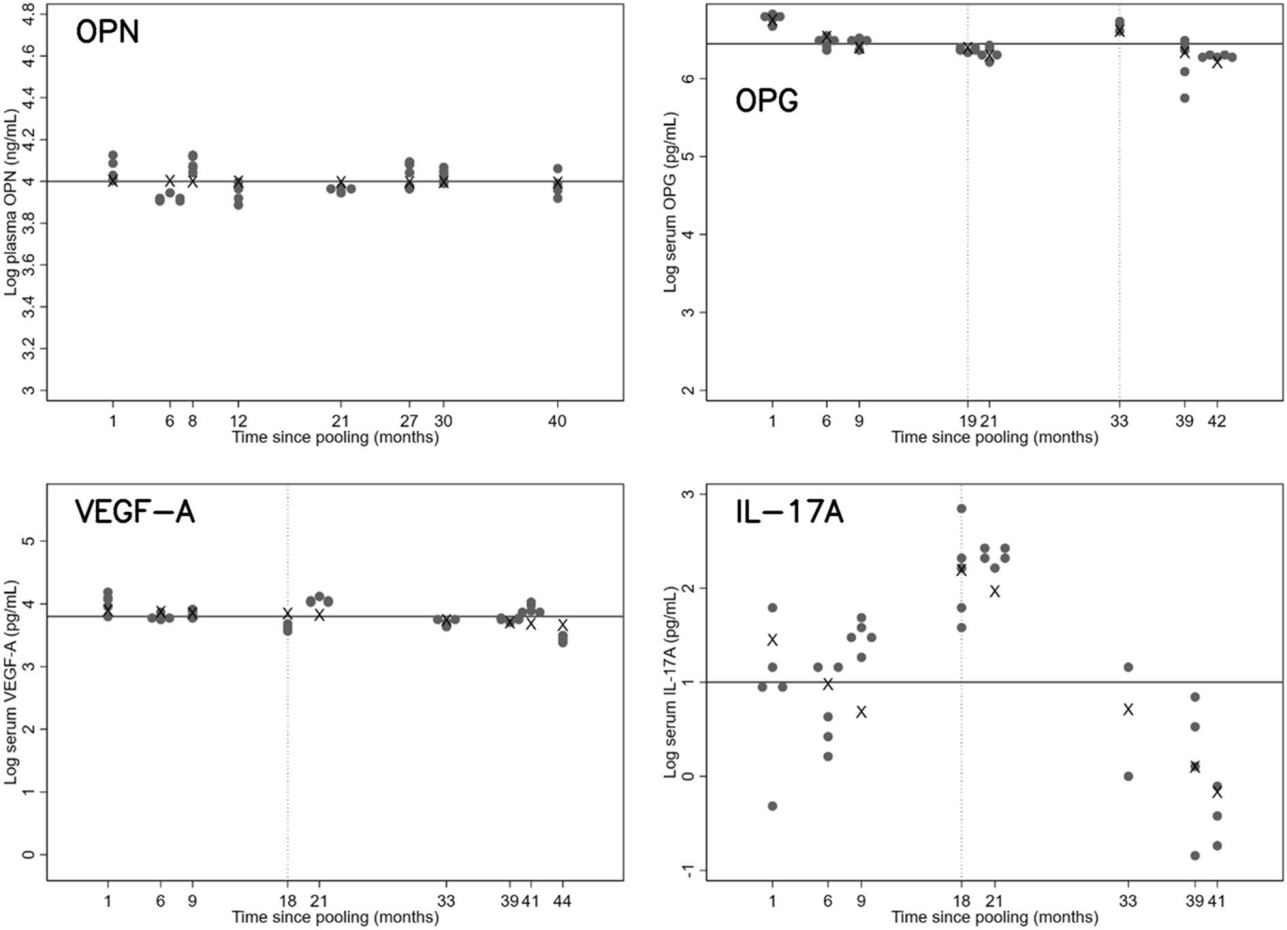
Distributions of measured values of cytokines in multiple aliquots of a pooled sample after various durations of storage. The y-axes, except for IL-17A, are scaled to the ranges from the healthy volunteer measurements. The horizontal line is at the overall mean; the marks (×) indicate the estimated assay means from the mixed model regression. The left and right vertical lines in the OPG plot indicate the changes in the platform software and kit, respectively. The vertical line in the VEGF-A and IL-17A plots indicates the change in software. There was no change in the assay environment for OPN

There was no evidence of instability in OPN measurements during long-term storage whereas there was evidence of substantial decay (− 33% per year 95% CI [− 54, − 3.7]) in OPG measurements (Table 2). There was no evident decay in VEGF-A measurement during long-term storage, although the confidence interval suggested slight decay (Table 2). Intra- and inter-assay CV of OPN and OPG over a long period were good, and comparable to those over a short period. The intra-assay CV of VEGF-A was good, and comparable to that in the reliability assay. Inter-assay CV of VEGF-A was within an acceptable range but slightly higher than that in the short-term reliability assay. IL-17A showed significant decay, − 70% per year, during storage. In addition, both the inter- and intra-assay variability were larger than those over the short term (Fig. 2).

**Table 2:** Stability and reliability during long-term storage

	% change per year [95% CI]	Intra-assay CV [95% CI]	Inter-assay CV [95% CI]
OPN	− 0.3% [− 4.2, 3.8]	0.041 [0.032, 0.053]	0.059 [0.032, 0.11]
OPG^a^	− 33% [− 54, − 3.7]	0.12 [0.095, 0.16]	0.076 [0.022, 0.26]
VEGF-A^b^	− 6.3% [− 14, 1.8]	0.069 [0.055, 0.088]	0.20 [0.11, 0.36]
IL-17A^b^	− 70% [− 83, − 48]	0.85 [0.37, 2.91]	0.43 [0.36, 0.52]

Based on a mixed model, estimated by REML method, with a fixed effect of storage duration:

^a^Adjusted for the fixed effects of measurement environment (combination of kit and platform software) and ^b^ adjusted for the fixed effect of platform software

### Variation within individuals

**Fig. 3:**
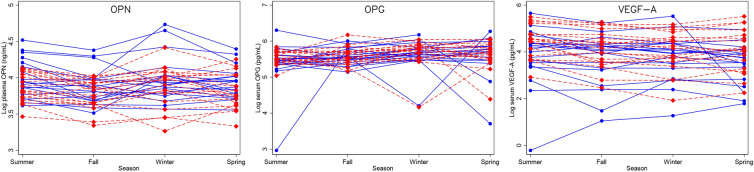
Seasonal values of measured circulating levels of cytokines within individuals. Males are shown as blue circles with solid lines, females as light red diamonds with dashed lines

Over the four seasons in a year, variation within individuals was small for OPN and VEGF-A. The ICC was good (0.68) for OPN and excellent (0.83) for VEGF-A (Table 3). The fourth measurement of VEGF-A from one individual was a suspected outlier (Fig. 3) because it was dramatically lower than the other values from that donor, and when this person was excluded, the ICC was 0.90. The intra-individual variation of OPG was high (ICC 0.11) as suspected from the crisscrossed trajectories (Fig. 3). Although the plot may give the impression that the low ICC resulted from a few potential low outliers, that was not the case; the trajectories of the non-outliers were still crisscrossed, and the ICC was still poor (0.31) after five potential outliers (those with OPG values less than 100 pg/mL) were excluded. Variation of IL-17A was not assessed because of large inter-assay variability.

**Table 3:** Intra-individual variation of the cytokines

	Intra-class correlation (ICC) [95% CI]
OPN	0.68 [0.55, 0.80]
OPG	0.11 [0.021, 0.43]
VEGF-A	0.83 [0.73, 0.90]

## Discussion

We found small intra- and inter-assay variability of OPN, OPG, and VEGF-A over short and long periods. This reliability makes these cytokines usable as biomarkers in a large population study. In contrast, IL-17A had large variability both within and between assays. Although one could overcome the variability within assays by measuring the same sample multiple times, the variability between assays is unsurmountable. This variability suggests that usability of IL-17A is limited to a single assay that measures the same samples repeatedly. The overall results of our study illustrate the need for validation under relevant conditions when measuring circulating cytokines in population studies.

We also found that the circulating levels of OPN and VEGF-A within individuals remained stable over a year, showing for the first time the intra-individual stability of serum OPN levels and confirming the previously reported intra-individual stability of serum VEGF-A levels [15–18]. This stability, along with the measurement reliability, allows these two cytokines to be measured in large population studies or to serve as potential biomarkers. On the other hand, the serum level of OPG fluctuated substantially within each individual. This variation could limit its usefulness as a biomarker and might have contributed to the inconsistency in its reported associations with disease [3, 13].

We did not assess intra-individual variation of serum IL-17A because of its inter-assay variability, a practical decision similar to that made in a study by Belzeaux et al. [15]. On the other hand, Guo et al. [16] showed good intra-individual correlation of IL-17A by canceling out inter-assay variability through experimental design—measuring the samples from the same person on the same plate. Although their experimental design was sophisticated, we do not regard such observed good correlation to be applicable in population studies given the large inter-assay variability and the difficulty of adhering to such a rigid design in practice.

A caveat should be made in regard to VEGF-A. Although it has small measurement variability and intra-individual variation, our results suggest that inter-assay variability increases over long-term experiments. This increase may have resulted from differences in experimental conditions, such as multiple reagent lots. In a large-scale study over a long period, investigators may have to pay attention to the possible worsening of inter-assay reliability with time.

Large cohort studies may require long-term sample storage, raising a concern about potential instability arising with storage [21]. In this study, we showed that this is not the case for OPN. We also showed no practically significant instability of VEGF-A, which is consistent with the report by Hetland et al. [17], although the possibility of slight decay remains. In contrast, we found substantial degradation of OPG during storage, as was reported by Chan et al. [19]. This instability could further complicate its measurement in cohort studies. Our experiment has shown that degradation of OPG during storage could be up to − 54% per year, or − 6.2% per month (upper limit of the 95% CI). When the time interval of sample storage in a study is restricted to a few months, the effect of degradation may remain relatively small compared to the assay variability. However, as storage time interval increases, the effect may become greater and require some adjustment in the analyses, even if the sample storage can be considered randomly distributed. It is also important to note that the relative impact of degradation would depend on the degree of inter-individual variation, or effect size, in the population.

Our study has several limitations. First, except for OPN, we assessed storage stability using different assay kits and platform software because of changes imposed by the manufacturer. Although we confirmed comparability between the environments for VEGF-A, we could not do so for OPG, and we had to adjust for the different environments in the analysis. This could have introduced bias in the assay for the storage stability of OPG. Second, the assays for intra-individual variation were conducted in batches of samples from the same seasons. Ideally, the experiment should have been designed so that individuals, seasons, and batches were orthogonal, but limitations on the allocation of human and laboratory resources for experimental assays in favor of conducting clinical examinations at RERF forced us to implement the current simpler design. This design could have introduced confounding between inter-assay variability and stability during storage, thereby leading to over-estimation of the intra-individual variation, or less likely, to under-estimation by incidentally canceling out the effect of storage time (if any). Third, the storage stability assay used re-frozen samples, which might have complicated the experiment because repeated freeze–thaw cycles could affect measurement [22]. However, we do not believe this additional freeze–thaw cycle introduced bias because the thawed samples were pooled so that all the subsequent subsamples had undergone the same number of freeze–thaw cycles. In addition, previous studies on VEGF-A [17, 23], OPN [24], or OPG [25], have reported no evidence of instability from a single refreeze–thaw cycle. Finally, our samples had cytokine levels in the middle of the measurable ranges. The reliability could be worse at the extremes of the measurable ranges.

## Conclusions

Our study showed that OPN and VEGF-A can be reliably measured in a large population but that IL-17A is suitable only for small experiments. For OPG, our results suggest that investigators should pay attention to intra-individual variation and potential degradation during storage.

### Supplementary Information

**Additional file 1.** Comparison of software or kits for VEGF-A and OPG measurement.

## Data Availability

The datasets used during the current study are available from the corresponding author upon reasonable request.

## Abbreviations

OPN: Osteopontin
OPG: Osteoprotegerin
VEGF-A: Vascular endothelial growth factor A
IL-17A: Interleukin-17A
CV: Coefficient of variation
RERF: Radiation Effects Research Foundation
AHS: Adult Health Study
ICC: Intra-class correlation
