# Supplementary material for: Reliability, stability during long-term storage, and intra-individual variation of circulating levels of osteopontin, osteoprotegerin, vascular endothelial growth factor-A, and interleukin-17A
Source: Eur J Med Res. 2024 Feb 17;29:133. doi: 10.1186/s40001-024-01722-w (PMC10873926; doi:10.1186/s40001-024-01722-w)
Supplement: Supplementary file 1 — Additional file 1. Comparison of software or kits for VEGF-A and OPG measurement. [file 40001_2024_1722_MOESM1_ESM.docx]

**Supplementary materials**


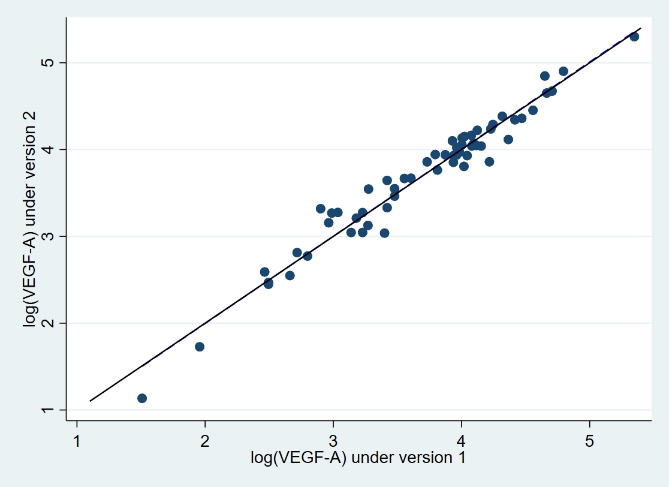

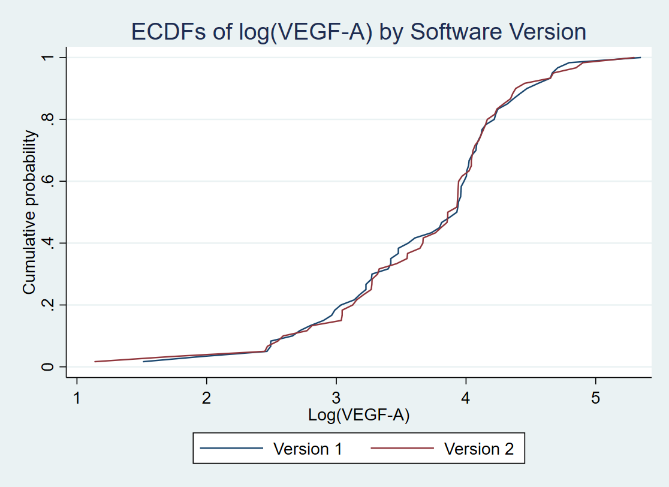


**Figure S1** Comparison of VEGF-A measurement between the older (DNASISPlex) and updated (Luminex xPOTENT) platform software, designated as version 1 and 2, respectively. Samples from 60 AHS participants were measured twice in two batches each with older and updated software, respectively. The latter assay was performed 7 months later than the former by using the same reagent lot.　A) The points on the scatter plot mostly fall close to the line of identity (the solid black line), reflecting similarity between the distributions of the two sets of measurements. The dashed line represents the regression line. B) Almost identical empirical cumulative distribution functions of VEGF-A measurements with older and updated platforms


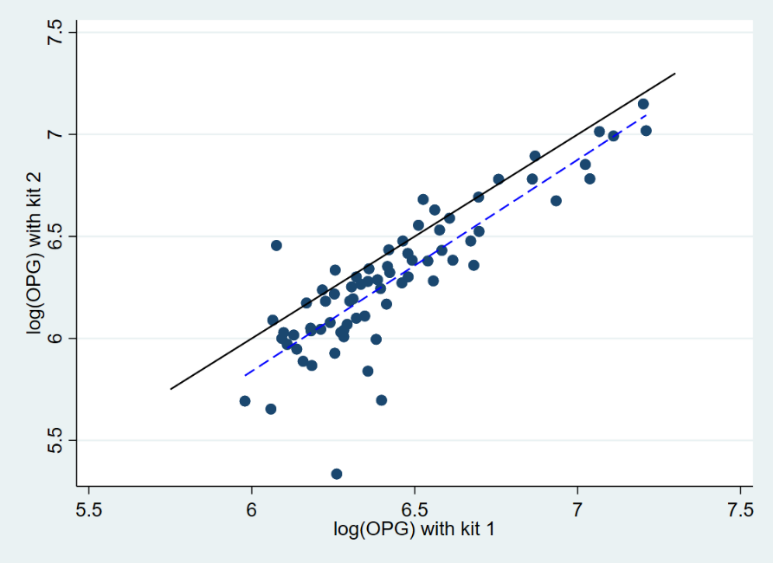


**Figure S2** Comparison of OPG measurement between older and updated assay kits. A total of 73 samples from AHS participants were measured twice, one week apart, first with the older kit (kit 1, polystyrene beads) and then with the updated kit (kit 2, magnetic beads). Each kit-specific assay was performed in a single batch. The points on the scatter plot mostly fall below the line of identity (the solid black line), reflecting higher values in the first assay. The dashed line represents the regression line.
